#  Evaluation of the Analgesic Efficacy of Melatonin in Patients Undergoing Cesarean Section Under S pinal Anesthesia: A Prospective Randomized Double-blind Study

**Published:** 2016

**Authors:** Marzieh Beigom Khezri, Morteza Delkhosh Reihany, Sonia Oveisy, Navid Mohammadi

**Affiliations:** a *Qazvin University of Medical Science, Department of Anesthesiology, Faculty of Medicine, Shahid Bahonar Ave, PO Box 3419759811, Qazvin, Iran.*; b *Department of Anesthesiology , Faculty of Medicine, Qazvin University of Medical Sciences. *; c *Community and preventive medicine, Faculty of medicine, Qazvin University of Medical Sciences. *; d *Community and preventive medicine, Faculty of medicine, Iran University of Medical Sciences.*

**Keywords:** Melatonin, Analgesia, Spinal anesthesia, Cesarean section

## Abstract

Melatonin has been suggested as a new natural pain killer in inflammatory pain and during surgical procedures. We designed this randomized double-blind controlled study to evaluate the analgesic efficacy and also optimal preemptive dose of melatonin in patients undergoing cesarean section under spinal anesthesia . One hundred twenty patients scheduled for cesarean section under spinal anesthesia were randomly allocated to one of three groups of 40 each to receive melatonin 3 milligram (mg) (group M_3_), melatonin 6 mg (group M_6_) or placebo (group P) sublingually 20 min before the spinal anesthesia. The time to first analgesic request, analgesic requirement in the first 24 h after surgery, hemodynamic variables, anxiety scores nd the incidence of adverse events were recorded. The duration of anesthesia and analgesia didn’t show significant differences between three groups. Total analgesic request during 24 h after surgery was different among the three groups (P = 0.035). The incidence of headache in group M_6_ was significantly higher than others (P<0.001). However, after adjusting headache between groups of the study, we were unable to show the significant difference in the total analgesic request during 24 h after surgery among the three groups (p = 0.058). Although premedication of patients with 3 mg sublingual melatonin prolonged time to first analgesic request after cesarean delivery compared to placebo group, the difference was not statistically significant. Meanwhile increasing dose of melatonin to 6 mg failed to enhance analgesia and also increase the incidence of headache in patients undergoing cesarean section under spinal anesthesia.

## Introduction

Postoperative pain has a major impact on the recovery process and patient satisfaction after Surgery (1). Good control of pain after cesarean section will improve mobility and can reduce the risk of thromboembolic events (2). Moreover, pain relief after cesarean delivery is associated with improved breastfeeding and infant rooming. Meanwhile, inadequate analgesia leads to elevated plasma catecholamine concentrations, which distress every organ system. The post-cesarean delivery pain is caused by surgical incision and uterus contraction with multiple mechanisms, which includes both central and peripheral sensitization, and also descending inhibition pathway (3). Therefore, non-steroidal anti-inflammatory agents (NSAIDs) and opioids play important roles in the management of pain. However, in parturient period, beneficial analgesia has to be balanced against known fetal and maternal effects, including respiratory depression, arterial hypotension, emetogenesis, pruritus, and direct and indirect irritation of the gastrointestinal tract (3). Such adverse effects may hamper the early post-operative recovery and quick breastfeeding and infant rooming. Therefore, the potential clinical advantages of new drugs in this setting remain to be evaluated. Melatonin or N-acetyl-methoxy tryptamine is mainly a neurohormone produced by the pineal gland. Melatonin has been suggested as a new analgesic drug and natural pain killer in inflammatory and neuropathic pain as well as surgical procedures (4, 5). It is reported that melatonin inhibits inflammation and tissue injury by affecting COX-2 and nitric oxide activity (4, 5, 6). The favorable role of melatonin in placental and fetal well-being is frequently reported (7, 8). Melatonin specifically distracts the circadian rhythms and promotes fetal growth and neurogenesis. Also, there is no evidence of unfavorable fetal or neonatal outcomes after its use (7, 8). In addition, the similarities between melatonin and oxytocin signaling might be leading to reduce blood loss via increased contractility of myometrium (9). Hence, these favorable effects may be valuable when melatonin is used as a premedication for cesarean section under spinal anesthesia. Use of melatonin as an anxiolytic agent has been studied extensively in human and animals (10-17). Although, there is evidence that in non-obstetric patients, melatonin decreases postsurgical pain, but the results of the previous studies are conflicting in gynecology/obstetrics. Also, an optimal melatonin dose with analgesic potentials is still unclear (11-18). According to the best of our knowledge, this study is the first in which the analgesic effect of melatonin in cesarean section has been evaluated. Our hypothesis was that melatonin may decrease the severity of pain after cesarean section without produce serious side effects apart from the anxiolytic effects during cesarean section under spinal anesthesia.

## Methods

**Figure 1:**
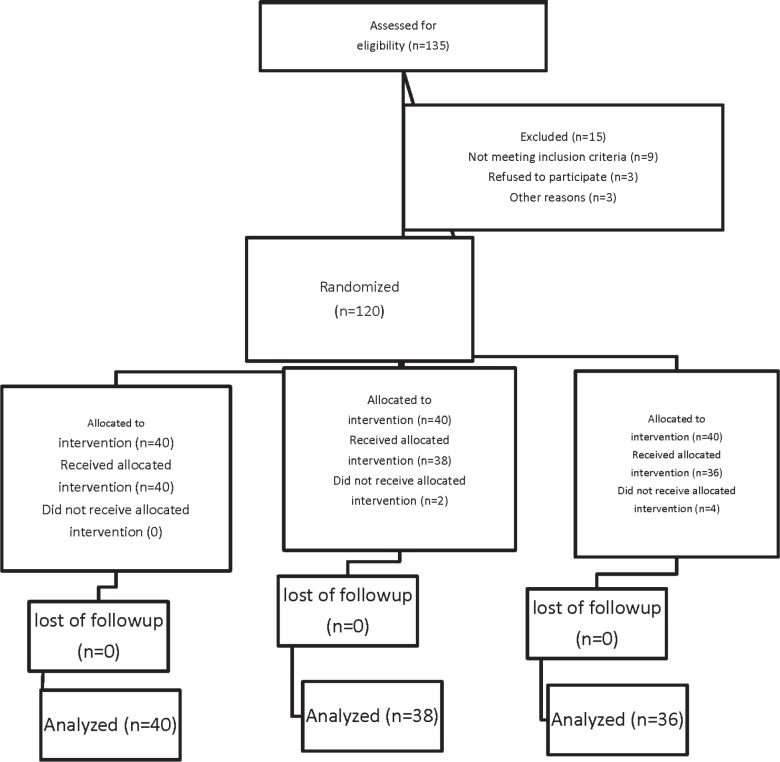
Consort flow diagram.

Following Ethics Committee approval and taking informed consents, we recruited 135 patients in a prospective, double-blind, randomized parallel trial in the Kowsar Hospital, Qazvin, Iran from April 2012 to October 2012. The patients were in a range of 18-40 year-old with an ASA physical status I or II who were scheduled for cesarean section under spinal anesthesia. The Consolidated Standards of Reporting Trials (CONSORT) recommendations for reporting randomized, controlled clinical trials were followed (Figure 1.) (19). Exclusion criteria included significant coexisting disease such as hepatorenal diseases, psychotic disorders, chronic cardiovascular disease, history of chronic headache, any contraindication to regional anesthesia such as local infection or bleeding disorders, allergy to melatonin, long-term antidepressant or analgesic drug use, any risk factor associated with an increased risk of postpartum hemorrhage such as multiple gestation, antepartum hemorrhage, polyhydramnios, and a history of previous rupture uterus. Patients were randomly allocated to one of three groups of 40 each to receive melatonin 3 milligram (mg) (Melatonin, Natural Webber; Canada) (group M3), melatonin 6 mg (Melatonin, Natural Webber; Canada) (group M6) or placebo (group P) sublingually 20 min before the spinal anesthesia. Randomization was undertaken by means of computer generated random number in sealed opaque envelops. Allocation was managed by a resident external to the project and the study drugs given by a nurse non–involved in the study. The anesthetist was blinded to the patient’s group assignment, and the study data were recorded by a blinded observer. No premedication was given except for the drugs predetermined by the study protocol. All patients received an intravenous preload of 5-7 mL/Kg lactated Ringer’s solution before a subarachnoid block. After using an aseptic technique, a 25-gauge Quincke needle was inserted intrathecally via a midline approach into the L4-5 interspaces with the patient in the sitting position by the same resident. Immediately after delivery of the neonate, intravenous infusion of oxytocin (20 IU syntocinon dissolved in 0.5 liter of lactated Ringer’s solution) over a 15 min period, was administered. Additional oxytocin (2.5 IU) was bolus injected if the surgeon considered uterine tone to be inadequate. At the preoperative visit, the verbal anxiety score (VAS) ranging from 0 to 10 (0= completely calm, 10= the worst possible anxiety) were explained to patients. The verbal anxiety score (VAS) ranging of patients was assessed at before premedication, before spinal anesthesia, 5 min after spinal anesthesia, after delivery of neonate, 15 min after spinal anesthesia, and after the surgery at recovery room. The primary outcomes of this randomized, double blinded placebo-controlled clinical trial were to evaluate the time to first requirement of analgesic supplement and total analgesic consumption in the first postoperative 24 h. The duration of spinal anesthesia was defined as the time from spinal injection to the first occasion when the patient complained of pain in the post-operative period. Also, the postoperative analgesia was defined as the time to the first requirement of analgesic supplement from the time of injection. No additional analgesic was administered unless requested by the patient. Patients were preoperatively instructed to use the verbal pain scale (VPS) from 0 to 10 (0: no pain, 10: maximum imaginable pain) for pain assessment. If the VPS exceeded four and the patient requested a supplement analgesic, a suppository (100 mg) of sodium diclofenac was given to relieve the post-operative pain as needed. For breakthrough pain (VPS >4) if the time for administration of diclofenac Na decreased to less than 8h, pethidine 25 mg was given intravenously (IV). The secondary outcomes of this study included the assessment of hemodynamic variables, anxiety scores, the incidence of hypotension, ephedrine requirements, bradycardia, hypoxemia [saturation of peripheral oxygen (SpO2) <90], adverse events such as nausea and vomiting, headache and APGAR score of neonates. 

Based on the data from previous similar studies, a sample size of 28 patients per group was required to detect a 20-min difference in the mean duration of analgesia between the groups with β = 0.1, and α = 0.05(11-14). We included 40 patients in each group to allow for probable dropouts and protocol violations. Data were analyzed using SPSS (SPSS 15.0, SPSS Inc, Chicago, IL, USA). Continuous variables were tested for normal distribution by the Kolmogorov–Smirnov test. Normally distributed data were expressed as means and standard deviations (SD). Analysis of variance (ANOVA)and repeated measures analyses were used for continuous parametric variables. Within groups comparisons were made using the LSD Post-hoc analysis. Chi square and Fisher›s exact tests were used for comparison the incidence of side effects between the groups. A pvalue<0.05 was considered as significant, statistically.

## Results

Of total one hundred thirty five patients initially enrolled in this study, 15 patients had to be excluded because of logistical reasons or other violations of the study protocol. One hundred twenty patients were included and randomly assigned to their treatment groups (Figure 1). 

No significant differences in age, stature, and weight among the three groups were found. The duration of surgery was also similar (Table 1.).

**Figure 2:**
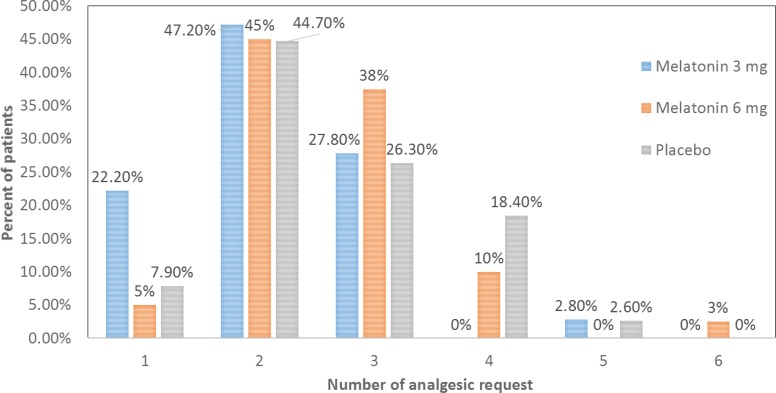
Comparison of total number of analgesic request in the first 24 h postoperative in the three groups.

**Figure 3:**
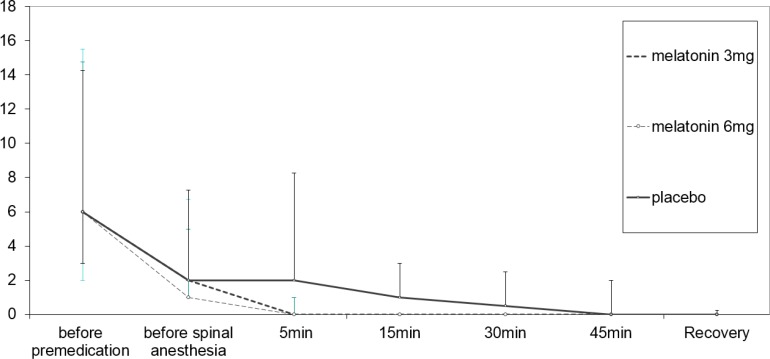
Comparison of Anxiety Scores in three study groups.

**Table 1:** Demographic data associated with the study groups

Groups	**P** **(N=38)**	M_6_ **(N=40)**	M_3_ **(N=36)**	**P**_Value_
Weight (kg)	75.21±7.15	72.45±6.59	74.75±6.88	0.169
Height (cm)	161.6±3.78	160.98±3.17	160.56±3.18	0.409
Age (years)	28.63±5.31	28.38±5.67	28.19±6.21	0.947
Duration of surgery (min)	85.63 ± 15.70	79.16±20.11	81.70±18.76	0.840

Values are presented as mean±SD.

**Table 2:** Comparison of analgesic duration in three study groups

**P**_value_	**M3** **(N=36)**	**M**_6_ **(N=40)**	**P** **(n=38)**	**Groups**
0.090	83.47±26.26*	79.77±25.83	70.68±24.77	ِDuration of spinal anesthesia (min)
0.076	208.19±122.66*	196.82±127.25	152.13±79.4	Time to first analgesic request

**Values are presented as mean±SD.**

* **As mentioned in the text, P values from LSD test of Post Hoc analysis in M3 versus P groups were significant.**

**Table 3:** Side effects observed in three study groups

**Groups**	**P** (n=38)	**M**_6_ (n=40)	**M**_3_ (n=36)	**P**_value_
Pruritus	0	2(%5)	0	0.152
Respiratory depression	0	0	0	1.000
Nausea	20 (%52.6)	17 (%242.5)	13 (%36.1)	0.351
Vomiting	12 (%31.6)	12 (%30)	5 (%13.9)	0.155
Headache	4(10.5%)	18 (%45)	2(5.6%)	<0.001
Vertigo	0	3 ((%7.5)	0	0.058

**Data are presented as number of patients (%). P=placebo, M**_3_ **=Melatonin 3mg, M**_6_ **=melatonin**

**6 mg.**

**Table 4:** Changes in hemodynamic variables

Groups	**P** **N=36**	**M**_6_ **N=40**	**M**_3_ **N= 36**	**P **_value_
variation of MAP	44.5±14.27	46.37±14.54	43.66±11.05	0.063
variation of HR	36.73±12.82	41.02±16.36	42.03±16.92	0.712

Values are presented as mean ± SD, MAP = mean arterial blood pressure (mmHg); HR = heart rate (bpm). P = placebo, M_3_=Melatonin 3 mg, M_6 _= melatonin 6 mg. The mean variation of MAP and HR was defined as the difference between the highest and the lowest mean arterial pressure and heart rate in each patient.

Table 2. shows that mean time of duration of anesthesia was longer in group M_3_ (83.47 ± 24. 77) than in groups M_6_ (79.72 ± 25.83) and P (70.68 ± 24.77min), but the difference between three groups were insignificant. Meanwhile, as shown in Table 2. the mean time to first analgesic request was also longer in group M_3 _(208.19 ± 122.66) than in groups M_6_ (196 ± 127.25) and P (152.13 ± 79.4 min), but the difference between three groups were not significant.

The difference of total number of analgesic request by patients during 24 h after surgery among three groups were significant (P = 0.035). The total of analgesic request by patients during 24 h after surgery was significantly lower in M_3_ group compared to P group (P = 0.024) and M_6_ group (P = 0.024) while there wasn’t any difference between M6 and placebo groups (P = 0.975) through LSD post hoc test. Figure 2. shows that the frequency of patients who requested analgesic at least four times was greater in placebo compared to M_3_ group. On contrary, the frequency of patients who requested analgesic only once was greater in groupM_3_ compared to other two groups.

As shown in Table 3. apart of headache, no significant differences were found in the three groups in terms of other intraoperative and postoperative side effects including pruritus, nausea, vomiting, vertigo and respiratory depression. The incidence of headache in group M_6_ was significantly higher than other groups (P<0.001).

After adjusting headache between groups of the study, we were unable to show the observed difference in the total number of analgesic request by patients during 24 h after surgery any more among the three groups (p = 0.058).

As shown in Figure 3. comparison of anxiety scores between three groups revealed significant difference through repeated measures analysis (P = 0.024). The anxiety score decreased significantly in the group of 6 mg melatonin compared to the placebo group through a repeated measures analysis and Scheffe’s post hoc test (p = 0.043).

Transient hypotension occurred at various time points in three groups, despite pre-block volume loading. These patients were treated with 5mg of ephedrine (boluses IV) to maintain systolic blood pressure (SBP) within 20% of baseline values or 90 mmHg. Comparison of mean arterial pressure (MAP) (P = 0.547) and heart rate (HR) (P = 0.227) changes during spinal anesthesia and surgery failed to reveal any statistically significant differences between all groups through repeated measure analysis. The mean variations of MAP and HR were defined as the difference between the highest and the lowest mean arterial pressure and heart rate in each patient and compared between the groups. Table 4. shows that difference of MAP variation between three groups were insignificant (P = 0.063) as well as the overall difference in ephedrine requirement (P = 0.239). Also, as shown in Table 4. the difference of mean HR variation between three groups were insignificant (P = 0.712).

All newborns in our study were free of any adverse effect. The Apgar scores at first (P_ = _0.212) and five (P_ = _0.367) minutes after delivery in the three groups were statistically similar. 

## Discussion

Based on the data found in our study, we concluded that although premedication of patients with 3 mg sublingual melatonin prolongs time to first analgesic request after cesarean delivery compared to placebo group but the difference was not statistically significant. Meanwhile increasing dose of melatonin to 6 mg failed to enhance analgesia and also increase the incidence of headache in patients undergoing cesarean section under spinal anesthesia. Our results with regard to the analgesic effect of melatonin are consistent with some previous studies (15-18). The findings of our previous studies and two studies carried out by Naguib *et al*. also indicating that there is no significant difference in the intraoperative opioid use or total doses of analgesics consumption in the melatonin or placebo groups over ninety min after the end of anesthesia at post-anesthesia care unit (15, 17). Yousaf *et al*. also in a systematic analysis of qualified clinical trials suggested that the analgesic effects of melatonin during the perioperative period is limited and results remain controversial (18). However, several previous studies reported that pain scores in the melatonin group, were significantly lower than in the control group. Anywise, these apparently controversial findings may be due to either the difference in melatonin doses or dissimilarity in population and the kind of surgeries, anesthesia (11, 14) .However the analgesic properties of melatonin have been shown to depend on the inhibition of inflammatory pathway and tissue injury by affecting COX-2 and nitric oxide activity (20, 21). Furthermore there is evidence that melatonin modulates glutamatergic systems involve the NMDA receptor Some results of present study are also supported by recent studies which suggested that melatonin can display its antinociceptive effects through indirect interacting with a number of neurotransmitter systems including benzodiazepinergic receptor, opioidergic and sigma receptor, serotonergic, dopaminergic, adrenergic, glutamatergic (NMDA receptor), NO-cyclic GMP-PKG signaling pathway, and directly through melatoninergic MT_1_/MT_2_ receptors (21-23). We chose to administer melatonin sublingually 20 min before the surgery in our study because the onset of melatonin-induced sedation has been reported to appear approximately 20-30 min after administration and the melatonin concentration remains stable for approximately 1.5 h at its peak concentration (24). Since the optimal effective analgesic dose of melatonin is still unclear, we chose to administer 3 and 6 mg of melatonin because these are the most commonly used dose of melatonin in both acute and chronic pain studies The next observation of study which should be noted is that the total of analgesic request by patients during 24 h after surgery was significantly lower in M_3_ group compared to other groups while there wasn’t any difference between M_6 _and placebo groups (18). Despite to the previous study in rats by Yu, Zhu *et al*., which reported that melatonin produces dose-dependent antinociceptive effects (21, 25). we could not demonstrate the dose-dependent antinociceptive effects for melatonin. However, in other previous studies, a similar hypnotic effect for melatonin at the dose between 0.3 and 10 mg was reported, a finding partially in accordance with that obtained in our study (26, 27). In present study increasing dose of melatonin to 6 mgnot only failed to enhance analgesia but also increased the incidence of headache in patients undergoing cesarean section under spinal anesthesia compared to M_3_ and control group. However, this finding indicates that sublingual melatonin could produce minor analgesic effects. The other possible explanation for this finding could be simultaneous application of spinal anesthesia and high dose melatonin may be associated with an increase in headache prevalence and thereby it induced shortening the time to first analgesic request in this group. In this study, after adjusting headache between groups of the study, we were unable to show the observed significant difference. Anywise we cannot offer a satisfactory good explanation for this finding and future studies are needed. 

In present study, despite the fact that a beneficial effect in treating migraine and cluster headache for melatonin in the several studies (28, 30). were reported, we observed the higher incidence of headache in patients who received 6mg melatonin. Peres *et al*. declared that a number of mechanisms like free radical scavenging, anti-inflammatory effects, inhibition of nitric oxide activity and dopamine release, GABA potentiation and neurovascular regulation, have been responsible for the favorable effects of melatonin in the treatment of cluster headaches and migraine attacks (28-30). However, there is general agreement that postpartum headache is a common troublesome complaint that can be worsened or caused by several factors ranging from hormonal shifts, physiological changes and peripartum procedures (31). In present study we excluded patients who had history of primary chronic headache. Since there is general agreement that the occurrence of PDPH (postdural puncture headache) following spinal anesthesia is influenced to some degree by anesthetic technique, with atraumatic (pencil-point) and smaller sized needles, these factors were controlled among the treatment groups in our study (32-34). In present study spinal anesthesia was performed by the same resident of anesthesiology in one technique which was described in the method section. The authors of the present study speculate that the high dose of melatonin may be augment the headache due to low cerebrospinal fluid (CSF) pressure and intracranial hypotension induced by the dural puncture. Dehghan *et al*. declared that daily administration of melatonin for 72 h after TBI (Traumatic Brain Injury) is effective in decreasing ICP and brain edema and improving neurological scores (34). According to another studies, melatonin therapy causes brain edema reduction and oral administration of melatonin 1 h prior and 1 h after ischemia induction in rats decreases brain edema (36, 37). Nevertheless, we cannot offer adequate explanation for this finding and further studies in large and different population are needed. Future studies are necessary to evaluate the analgesic efficacy of melatonin in patients undergoing cesarean section under general anesthesia.

## Conclusion

Based on the study, we concluded that premedication of patients with 3 mg sublingual melatonin provided minor analgesic effects compared to placebo group. Meanwhile increasing dose of melatonin to 6 mg failed to enhance analgesia and also increase the incidence of headache in patients undergoing cesarean section under spinal anesthesia.

## References

[1] Breivik H, Stubhaug A (2008). Management of acute postoperative pain: still a long way to go!. Pain.

[2] Gadsden J, Hart S, Santos AC (2005). Post-cesarean delivery analgesia. Anesth. Analg.

[3] Wong JO, Tan TD, Cheu NW, Wang YR, Liao CH, Chuang FH, Watts MP (2010). Comparison of the efficacy of parecoxib versus ketorolac combined with morphine on patient-controlled analgesia for post-cesarean delivery pain management. Acta Anaesthesiol. Taiwan.

[4] Esposito E, Paterniti I, Mazzon E, Bramant P, Cuzzocrea S (2010). Melatonin reduces hyperalgesia associated with inflamm ation. J. Pineal Res.

[5] Srinivasan V, Zakaria R, Jeet Singh H, Acuna-Castroviejo D (2012). Melatonin and its agonists in pain modulation and its clinical application. Arch. Ital. Biol.

[6] Ghaeli P, Vejdani S, Ariamanesh A, Hajhossein Talasaz A (2015). Effect of melatonin on cardiac injury after primary percutaneous coronary intervention: A randomized controlled trial. Iran. J. Pharm. Res.

[7] Seron-Ferre M, Mendez N, Abarzua-Catalan L, Vilches N, Valenzuela FJ, Reynolds HE, Llanos AJ, Rojas A, Valenzuela GJ, Torres-Farfan C (2012). Circadian rhythms in the fetus. Mol. Cell Endocrinol.

[8] Nagai R, Watanabe K, Wakatsuki A, Hamada F, Shinohara K, Hayashi Y, Imamura R, Fukaya T (2008). Melatonin preserves fetal growth in rats by protecting against ischemia reperfusion-induced oxidative/nitrosative mitochondrial damage in the placenta. J.Pineal Res.

[9] Sharkey JT, Cable C, Olcese J (2010). Melatonin sensitizes human myometrial cells to oxytocin in a protein kinase C[alpha]/extracellular-signal regulated kinase-dependent manner. J. Clin. Endocrinol. Metab.

[10] Isik B, Baygin O, Bodur H (2008). Premedication with melatonin vs midazolam in anxious children. Paediatr. Anaesth.

[11] Ismail SA, Mowafi HA (2009). Melatonin provides anxiolysis, enhances analgesia, decreases intraocular pressure, and promotes better operating conditions during cataract surgery under topical anesthesia. Anesth. Analg.

[12] Caumo W, Levandovski R, Hidalgo M (2009). Preoperative anxiolytic effect of melatonin and clonidine on postoperative pain and morphine consumption in patients undergoing abdominal hysterectomy: A double-blind, randomized, placebo-controlled study. J. Pain.

[13] Mowafi HA, Ismail SA (2008). Melatonin improves tourniquet tolerance and enhances postoperative analgesia in patients receiving intravenous regional anesthesia. Anesth. Analg.

[14] Schwertner A, Conceição Dos Santos CC, Costa GD, Deitos A, de Souza A, de Souza IC, Torres IL, da Cunha Filho JS, Caumo W (2013). Efficacy of melatonin in the treatment of endometriosis: a phase II, randomized, double-blind, placebo-controlled trial. Pain.

[15] Khezri MB, Oladi MR, Atlasbaf A (2013). Effect of melatonin and gabapentin on anxiety and pain associated with retrobulbar eye block for cataract surgery: a randomized double-blind study. Indian J. Pharmacol.

[16] Khezri MB, Merate H (2013). The effects of melatonin on anxiety and pain scores of patients, intraocular pressure, and operating conditions during cataract surgery under topical anesthesia. Indian J. Ophthalmol.

[17] Naguib M, Samarkandi AH (2000). The comparative dose-response effect of melatonin and midazolam for premedication of adult›s patients: A double-blinded placebo-controlled study. Anesth. Analg.

[18] Yousaf F, Seet E, Venkatraghavan L, Abrishami A, Chung F (2010). Efficacy and safety of melatonin as an anxiolytic and analgesic in the perioperative period. Anesthesiology.

[19] Moher D, Schulz KF, Altman DG, CONSORT (2001). The CONSORT statement: revised recommendations for improving the quality of reports of parallel group randomized trial. BMC Med. Res. Methodol.

[20] Esposito E, Paterniti I, Mazzon E, Bramant P, Cuzzocrea S (2010). Melatonin reduces hyperalgesia associated with inflammation. J. Pineal Res.

[21] Srinivasan V, Lauterbach EC, Ho KY, Acuña-Castroviejo D, Zakaria R, Brzezinski A (2012). Melatonin in antinociception: its therapeutic applications. Curr. Neuropharmacol.

[22] Ambriz-Tututi M, Granodos-Soto V (2007). Oral and spinal melatonin reduces tactile allodynia in rats via activation of MT2 and opioid receptors. Pain.

[23] Mantovani M, Kaster MP, Pertile R, Calixto JB, Rodriguez AL, Santos AR (2006). Mechanisms involved in the antinociception caused by melatonin in the mice. J. Pineal Res.

[24] Markantonis SL, Tsakalozou E, Paraskeva A, Staikou C, Fassoulaki A (2008). Melatonin pharmacokinetics in premenopausal and postmenopausal healthy female volunteers. J. Clin. Pharmacol.

[25] Yu CX, Zhu CB, Xu SF, Cao XD, Wu GC (2000). The analgesic effects of peripheral and central administration of melatonin in rats. Eur. J. Pharmacol.

[26] Arendt J (2006). Melatonin and human rhythms. Chronobiol. Int.

[27] Gögenur I, Kücükakin B, Bisgaard T, Kristiansen V, Hjortsø NC, Skene DJ, Rosenberg J (2009). The effect of melatonin on sleep quality after laparoscopic cholecystectomy: a randomized, placebo-controlled trial. Anesth. Analg.

[28] Peres MFP (2005). Melatonin, the pineal gland and their implications for headache. Cephalagia.

[29] Peres MFP, Rozen TD (2001). Melatonin in the preventive treatment of chronic cluster headache. Cephalagia.

[30] Tabeeva GR, Sergeev AV, Gromova SA (2011). Possibilities of preventive treatment of migraine with MT1 and MT2 agonist and 5-HT2c receptor antagonist agomelatin (valdoxan). Zh. Nevrol. Psikhiatr. Im. S S Korsakova.

[31] Klein AM, Loder E (2010). Postpartum headache. Int. J. Obstet. Anesth.

[32] Evron S, Sessler D, Sadan O, Boaz M, Glezerman M, Ezri T (2004). Identification of the epidural space Loss of resistance with air, lidocaine, or the combination of air and lidocaine. Anesth. Analg.

[33] Chohan U, Hamdani GA (2003). Post-dural puncture headache. J. Pak. Med. Assoc.

[34] Kuczkowski KM (2004). Post-dural puncture headache in the obstetric patient: An old problem new solutions. Minerva. Anestesiol.

[35] Dehghan F, Khaksari Hadad M, Asadikram G, Najafipour H, Shahrokhi N (2013). Effect of melatonin on intracranial pressure and brain edema following traumatic brain injury: role of oxidative stresses. Arch. Med. Res.

[36] Bayir A, Kireşi DA, Kara H, Cengiz SL, Koçak S, Ozdinç S, Ak A, Bodur S (2008). The effects of mannitol and melatonin on MRI findings in an animal model of traumatic brain edema. Acta Neurol. Belg.

[37] Li ZQ, Liang GB, Xue YX, Liu YH (2009). Effects of combination treatment of dexamethasone and melatonin on brain injury in intracerebral hemorrhage model in rats. Brain Res.

